# Primary hepatic mucosa-associated lymphoid tissue lymphoma: a case report and literature review

**DOI:** 10.1186/s40792-015-0091-8

**Published:** 2015-09-24

**Authors:** Shigeyuki Nagata, Norifumi Harimoto, Kiyoshi Kajiyama

**Affiliations:** Department of Surgery, Iizuka Hosipital, Yoshiomachi 3-83, Iizuka, Fukuoka 820-8505 Japan

**Keywords:** Primary hepatic lymphoma, Mucosa-associated lymphoid tissue lymphoma, Hepatectomy, *Helicobacter pylori*

## Abstract

Primary hepatic mucosa-associated lymphoid tissue (MALT) lymphoma is an extremely rare disease. We herein describe the findings in a 74-year-old man with elevated liver enzyme levels. Dynamic computed tomography showed focal biliary dilation and atrophy in the posterior segment, while dynamic magnetic resonance images revealed a small, highly enhanced small mass located at the root of posterior branch of the biliary ducts. As the mass was not detected on abdominal ultrasonography, a biopsy could not be performed. Cholangiocellular carcinoma was suspected, and surgery was performed. However, the surgically resected hepatic tumor was a nodule of aggregated lymphocytes that formed a lymphoepithelial lesion. Immunohistochemical analysis revealed that the lymphoma cells were positive for CD20 and CD79a, but negative for CD3. No other lymphoid lesions were found during additional postoperative examinations. Therefore, the patient was diagnosed with primary hepatic MALT lymphoma. He was also diagnosed with *Helicobacter pylori* infection, and thus, pylorus eradication was performed. At the time of this report, the patient was free of disease for 2 years without any additional treatment. The present case contributed to the diagnosis and management of this rare disease, as previously published case reports described varying imaging features; it also suggested that preoperative diagnosis was often difficult without biopsy.

## Background

Mucosa-associated lymphoid tissue (MALT) lymphoma is a low-grade malignant lymphoma that was first described by Isaacson and Wright in 1983 [1]. The stomach is one of the most common sites of MALT lymphoma development, and gastric MALT lymphoma is commonly associated with *Helicobacter pylori* (HP) infection. However, primary hepatic lymphoma (PHL) is very rare, accounting for approximately only 0.016 % of all cases of all non-Hodgkin’s lymphoma cases [2]. Furthermore, primary hepatic MALT lymphoma is extremely rare among the diagnosed PHL cases. In addition, the standard diagnostic method and treatment strategy of this disease have yet to be established. Herein, we describe a case of surgically resected primary hepatic MALT lymphoma, which was initially suspected to be a cholangiocellular carcinoma, and review the relevant literature.

## Case presentation

A 74-year-old man was referred to our department for mild elevation of liver enzyme levels. He had no significant medical history except for hypertension that was medically managed. His family history was unremarkable. Physical examination at presentation did not indicate any abnormalities. The laboratory tests conducted at our hospital showed the following findings: hemoglobin level of 17.4 g/dl, a platelet count of 204,000/μl, albumin level of 4.5 g/dl, total bilirubin level of 0.6 mg/dl, aspartate aminotransferase level of 22 IU/L, alanine aminotransferase level of 34 IU/L, lactate dehydrogenase level of 160 IU/L, γ-glutamyltranspeptidase level of 36 IU/L, alkaline phosphatase level of 338 IU/L, C-reactive protein level of 0.43 mg/dl, IgG level of 2199 mg/dl, and IgM level of 268.7 mg/dl. Hepatitis B surface antigen and anti-hepatitis C virus antibody in the serum were negative. Anti-nuclear antibody and anti-mitochondrial antibody were also negative. Tumor marker levels including carcinoembryonic antigen, carbohydrate antigen 19-9, α-fetoprotein, and des-γ-carboxy prothrombin were within the normal ranges.

**Fig. 1:**
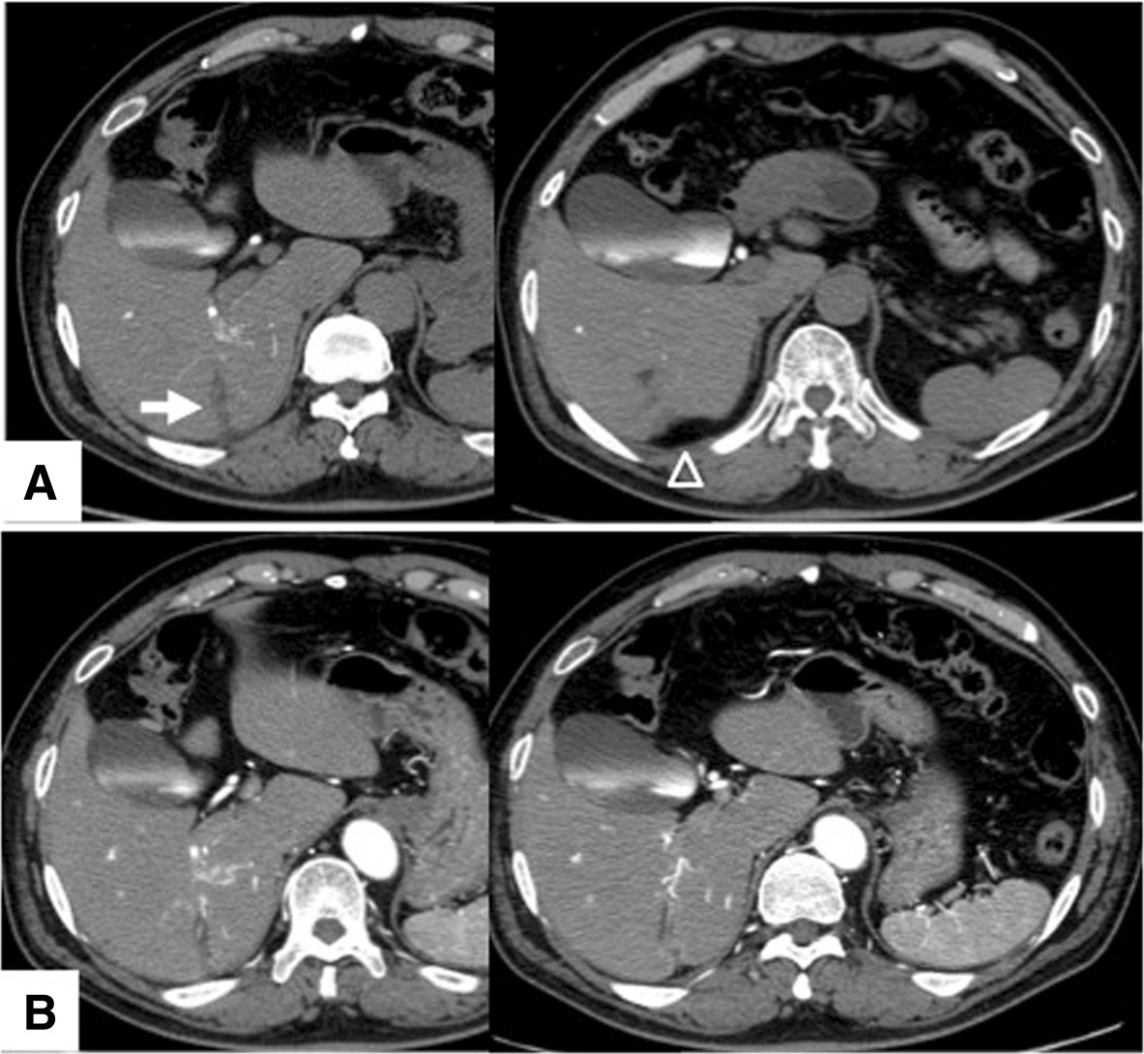
Computed tomography findings. **a** Dynamic computed tomography with drip infusion cholangiography revealed focal dilatation of the biliary ducts (*arrow*) and atrophy (*arrowheads*) in the posterior segments of the liver. **b** No tumor was detected via enhanced computed tomography

**Fig. 2:**
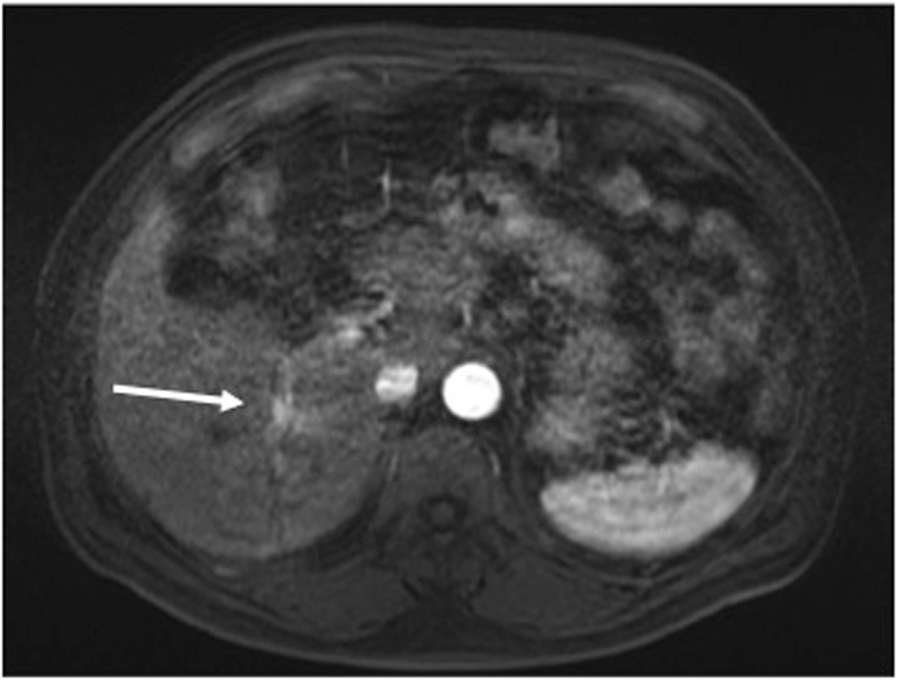
Enhanced magnetic resonance imaging after gadolinium injection. The tumor was hyper-intense on T1-weighted images (*arrow*) in the area adjacent to the main posterior biliary duct in the arterial phase but showed rapid washout in the late phase

**Fig. 3:**
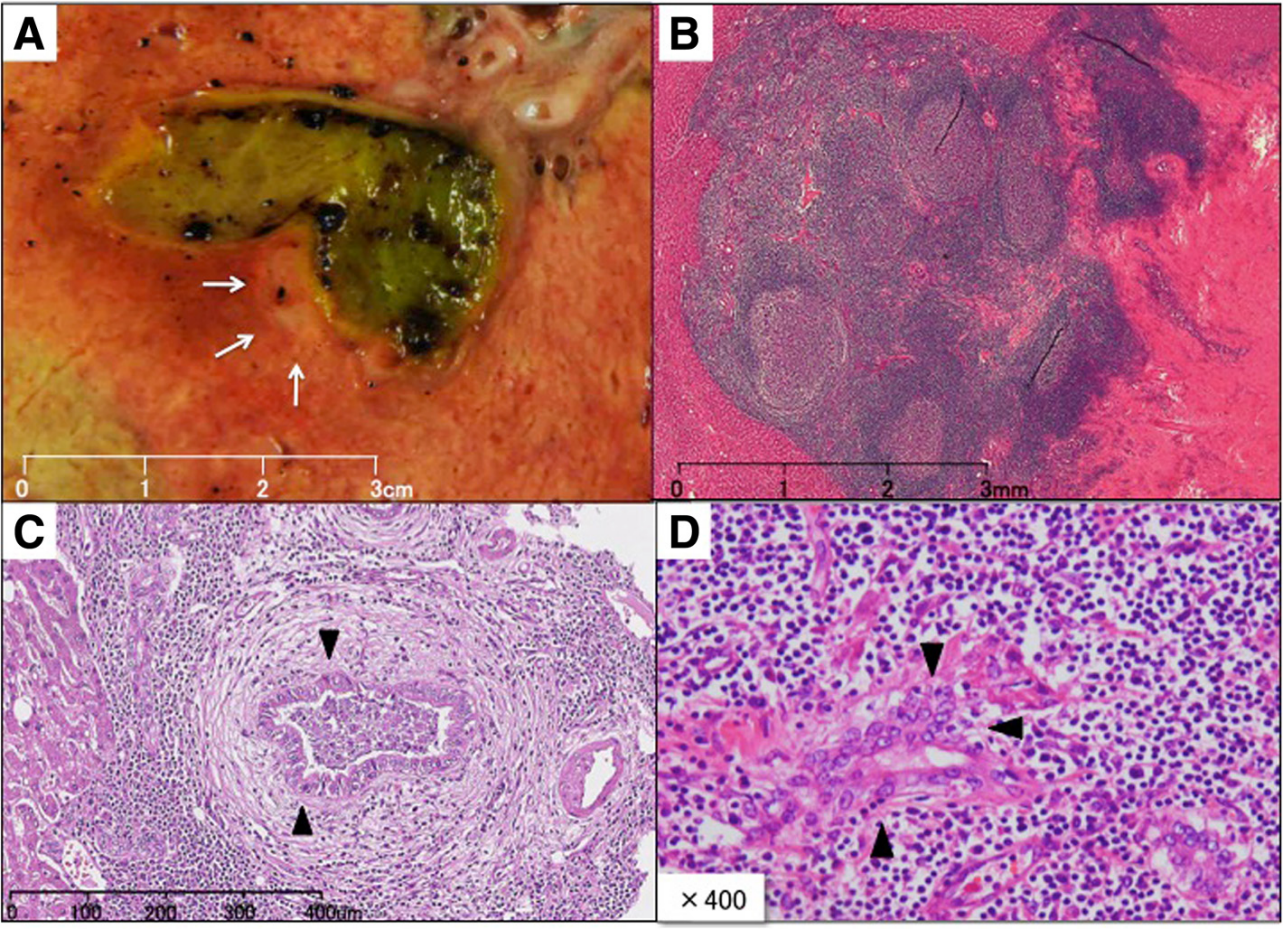
Tumor characteristics. **a** Grossly, the 7-mm white mass along the posterior biliary duct was soft and non-encapsulated. **b** Histological findings on hematoxylin and eosin staining. The lesion consisted of dense lymphocyte infiltration with some lymph follicles. **c** and **d** Small to mid-sized lymphocytes formed lymphoepithelial lesions on some bile capillaries

Dynamic computed tomography (CT) with drip infusion cholangiography revealed focal dilatation of the biliary ducts and atrophy in the posterior segments of the liver without any observable mass (Fig. 1a, b). The magnetic resonance imaging (MRI) scans, T1- and T2-weighted images, did not show any mass. However, when gadolinium was used as a contrast agent, a 1.5-cm mass located in the area adjacent at the main posterior biliary duct was highly enhanced on T1-weighted images during the arterial phase but demonstrated rapid withdrawal in the portal venous and delayed phases (Fig. 2). Gastroscopic and colonoscopic examinations showed no ulcerative or tumorous lesion. As the mass was not detected on abdominal ultrasonography (US) and it could possibly be a malignant tumor such as cholangiocellular carcinoma, the patient consented to *undergo* a right hepatectomy with lymph node dissection in the hepatic portal region. Grossly, a 7-mm white mass detected along with the posterior biliary duct was soft and non-encapsulated like a lymph follicle (Fig. 3a).

**Fig. 4:**
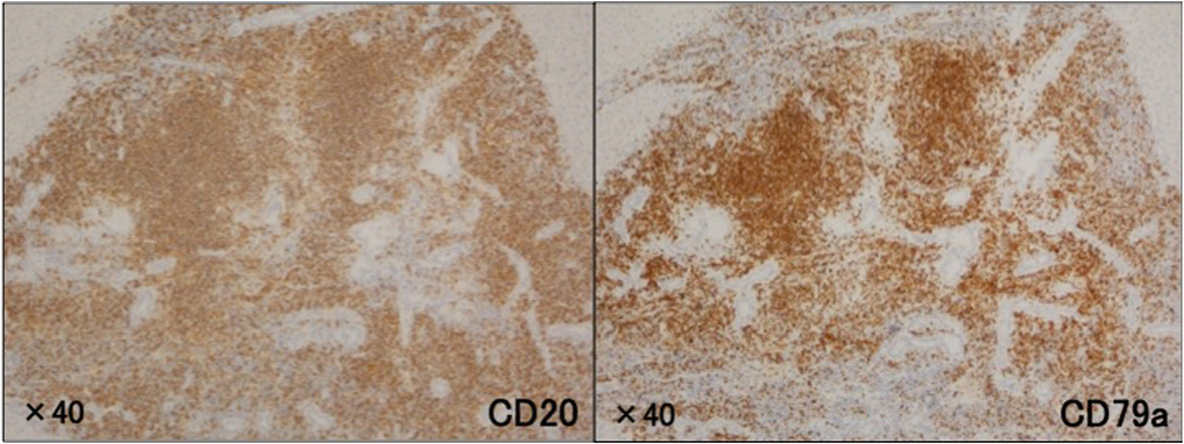
Histological findings by immunohistochemical staining. Lymphocytes were diffusely positive for CD20 and CD79a antibodies

Histologically, dense lymphocyte infiltration with some lymphoid follicles was observed in the portal area (Fig. 3b). Small- to middle-sized lymphocytes showed no apparent atypia but formed lymphoepithelial lesions on some bile capillaries (Fig. 3c, d). Immunohistochemical studies indicated that the lymphocytes were positive for CD20 and CD79a (Fig. 4), but negative for CD3. The patient was diagnosed with low-grade hepatic MALT lymphoma based on the abovementioned pathological findings.

Subsequently, the patient’s level of interleukin-2 receptor was found to be elevated at 1133 U/ml (normal range, 122–496 U/ml). He was also infected with HP and medical treatment for pylorus eradication was provided. Biopsy of the bone marrow revealed a normoplastic marrow. Positron emission tomography demonstrated diffuse accumulation in both the thyroid glands, with a maximum standardized uptake value of 4.0. Biopsy of the thyroid glands showed chronic thyroiditis without malignancy, and the patient’s thyroid function was within normal limits. The present case of MALT lymphoma was diagnosed a stage I tumor, according to the Ann Arbor classification, and careful follow-up without additional treatment was selected. At the time of this report, the patient remained alive and free of disease 2 years after surgery.

### Discussion

MALT lymphoma often develops at several anatomic sites, including the gastrointestinal tract, lungs, head and neck, skin, thyroid glands, breasts, and liver. Gastric MALT lymphoma is thought to be triggered by chronic inflammation, which can occur in different diseases including chronic gastritis associated with HP infection, Sjogren syndrome, and Hashimoto thyroiditis [3]. The etiology of primary hepatic MALT lymphoma is unclear, but it has been reported that primary biliary cirrhosis [4–8], hepatitis C viral infection [8–13], hepatitis B viral infection [14–16], ascariasis [17, 18], and HP infection [19] are possibly related with the pathogenesis of hepatic MALT lymphoma.

At presentation, our patient was not infected with hepatitis viruses, and his thyroid function and bone marrow were normal. He was also negative for anti-nuclear and anti-mitochondrial antibodies. However, his serum IgG and IgM levels were elevated, and he showed HP infection. Such clinical findings suggested that the hepatic MALT lymphoma might be strongly associated with chronic inflammation caused by HP infection. Subsequent treatment for HP infection after surgery was successful.

For literature review, we searched PubMed and Ichushi Web by Japan Medical Abstracts Society independently. Key terms used included “MALT lymphoma,” “liver,” “hepatic MALT lymphoma,” and “primary hepatic lymphoma. To our knowledge, there are 37 reports including 51 patients with primary hepatic MALT lymphoma [4–41] (Table 1). The mean age of these 22 men and 29 women was 64.0 years. In most cases, the hepatic tumors were incidentally detected during surgical resection or on follow-up imaging examination for liver diseases or other *conditions*. In 24 patients (47 %), liver diseases concomitantly existed (ascariasis, 2; primary biliary cirrhosis, 5; hepatitis B, 4; hepatitis C, 6; drug induced hepatitis, 1; cirrhosis without hepatitis viral infection, 5; and multiple biliary cysts, 1). Thus, hepatic MALT lymphoma development might be related to chronic liver inflammation, similar to gastric MALT lymphoma. Thirty-eight patients (74 %) had solitary mass, and the tumor size was ≤3 cm in 22 of the 41 reported cases (53 %). Regarding radiological characteristics, in 15 cases, the tumors were described as detectable hypo-echoic masses via abdominal US. In 21 cases, they were detected as low-density masses via CT, including 6 cases with enhancement and 9 without. In 16 cases with detailed MRI description, all tumors showed high density on T1-weighted images and low density on T2-weighted images. Two cases that described contrast-enhanced MRI showed sickly enhancement in the early phase. Both cases had solitary mass, and their tumor sizes were 3 and 6.5 cm, respectively [27, 41]. Our case showed highly enhanced mass in the early phase, but not detected in abdominal US and CT. It is suggested that these findings would be specific to small hepatic MALT lymphoma. With regard to treatment, 31 patients (60.8 %) underwent surgical resection with or without chemotherapy or radiation therapy. Of these, 28 patients had a single tumor, including 4 whose tumors were accidentally discovered in the isolated liver from transplantation patients. In addition, one patient underwent radiofrequency ablation, five received chemotherapy only, and two received radiation only. Eight patients did not receive any treatment, five of whom died during the follow-up period. Recurrence was reported in two patients.

**Table 1:** Reported cases of hepatic MALT lymphoma

Case	Sex/age	HBV	HCV	Concomitant disease	Tumor no.	Treatment	Outcome
1	M/66	ND	ND	Ureteral cancer	1	Resection	12 M/alive
2	F/73	ND	ND	(−)	1	Resection	Lost to follow-up
3	M/85	ND	ND	Prostatic cancer	2	(−)	Death after other surgery
4	F/60	ND	ND	Liver cirrhosis	Multiple	Transplantation	12 M/dead
5	F/57	(−)	ND	Ascariasis	1	Resection	55 M/alive
6	M/48	(+)	ND	Hepatitis	1	Resection + Chemotherapy	38 M/alive
7	F/47	(−)	(−)	Multiple biliary unilocular cysts	1	Resection + Radiation	30 M/alive
8	M/64	(−)	(−)	Colon cancer	1	Resection	Lost to follow-up
9	F/62	(−)	(−)	Primary biliary cirrhosis	1	Resection	6 M/alive
10	F/64	(−)	(+)	Liver cirrhosis	1	Chemotherapy	24 M/alive
11	F/65	(−)	(+)	Hepatitis	1	Chemotherapy	48 M/alive
12	F/69	(−)	(−)	(−)	1	Resection	Short time/alive
13	F/41	(−)	(−)	Primary biliary cirrhosis	1	(−)	12 M/alive
14	F/64	(−)	(−)	(−)	1	Resection	72 M/alive
15	F/57	(−)	(−)	Primary biliary cirrhosis	1	Transplantation	9 M/alive
16	F/64	(−)	(−)	Ascariasis	1	Resection	Pulmonary recurrence after 96 M
17	F/59	ND	ND	ND	Multiple	Resection + Chemotherapy	ND
18	M/61	(−)	(−)	Gastric cancer	1	Resection	18 M/alive
19	M/73	(−)	(+)	Liver cirrhosis	1	Resection	34 M/alive
20	M/59	(−)	(+)	Hepatitis	1	Resection	30 M/alive
21	F/50	(−)	(−)	(−)	1	Resection + Chemotherapy	30 M/alive
22	F/72	ND	ND	Colon cancer	1	(−)	1 M/dead
23	F/61	ND	ND	Rheumatoid arthritis	1	(−)	Dead
24	F/58	ND	ND	(−)	Multiple	Resection + Chemotherapy	37 M/alive
25	F/62	ND	ND	Breast cancer	1	Resection	9 M/alive
26	F/65	(+)	(−)	Hepatocellular carcinoma	1	Resection	10 M/alive
27	F/60	(−)	(−)	Gastric MALT lymphoma	1	(−)	30 M/alive
28	M/59	(+)	(−)	Liver cirrhosis	2	Transplantation	6 M/Alive
29	M/36	(+)	(−)	Hepatitis	1	Resection	Hepatic recurrence after 40 M
30	M/53	(−)	(+)	Liver cirrhosis	Multiple	Transplantation + Chemo	ND
31	M/67	(−)	(−)	Hepatitis (drug)	1	Radiation	Pulmonary recurrence after 72 M
32	M/69	(−)	(−)	(−)	2	RFA + Chemo	24 M/alive
33	F/74	(−)	(−)	(−)	1	Resection + Chemotherapy	6 M/alive
34	F/67	NA	NA	Gastric MALT lymphoma	Multiple	(−)	1 M/dead
35	F/72	(−)	(−)	Colon cancer	1	Resection	24 M/alive
36	M/64	(−)	(−)	Gastric cancer	Multiple	(−)	24 M/alive
37	M/71	(−)	(−)	(−)	1	Resection	15 M/alive
38	M/71	(−)	(−)	(−)	1	Resection + Chemotherapy	45 M/alive
39	F/56	(−)	(−)	(−)	1	Resection	Pulmonary recurrence after 84 M
40	M/59	(−)	(−)	(−)	1	Resection	5 M/alive
41	M/86	(+)	(−)	Hepatitis	1	(−)	15 M/alive
42	M/58	(−)	(+)	Hepatitis	1	Resection + Chemotherapy	6 M/alive
43	F/43	(−)	(−)	Gastric cancer	1	Resection	24 M/alive
44	F/80	(−)	(−)	Primary biliary cirrhosis	Multiple	Chemotherapy	ND
45	M/76	(−)	(+)	Hepatitis	1	Radiation	60 M/alive
46	F/74	(−)	(−)	Colon cancer	2	Resection	24 M/alive
47	F/74	(−)	(−)	Primary biliary cirrhosis	Multiple	Chemotherapy	36 M/alive, no relapse
48	M/73	(−)	(+)	Hepatitis	Multiple	Chemotherapy	24 M/alive, relapse
49	F/56	(−)	(−)	(−)	1	Resection	13 M/alive
50	M/77	(−)	(+)	Hepatitis	1	Resection	8 M/alive
Our case	M/74	(−)	(−)	(−)	1	Resection	30 M/alive

*ND* not detected

According to the abovementioned case reports, primary hepatic MALT lymphoma tends to be solitary and small. Furthermore, it is often difficult to make a definite diagnosis of primary hepatic MALT lymphoma solely based on the imaging findings as the disease seem to exhibit variable imaging features. Therefore, it is necessary to accumulate more cases and establish a therapeutic strategy for primary hepatic MALT lymphoma.

## Conclusions

In the present report, we described a case of primary hepatic MALT lymphoma. Our experience in this case and review of relevant literature indicated that preoperative diagnosis of hepatic MALT lymphoma might be challenging because of the disease’s varying imaging features. Thus, further study of this extremely rare disease is necessary.

## Consent

Written informed consent was obtained from the patient for publication of this case report and accompanying images.

## Abbreviations

CT: computed tomography
HP: *Helicobacter pylori*
MALT: mucosa-associated lymphoid tissue
MRI: magnetic resonance imaging
PHL: primary hepatic lymphoma
US: ultrasonography
